# Computational Hemodynamic Modeling of Arterial Aneurysms: A Mini-Review

**DOI:** 10.3389/fphys.2020.00454

**Published:** 2020-05-12

**Authors:** Sarah N. Lipp, Elizabeth E. Niedert, Hannah L. Cebull, Tyler C. Diorio, Jessica L. Ma, Sean M. Rothenberger, Kimberly A. Stevens Boster, Craig J. Goergen

**Affiliations:** ^1^Weldon School of Biomedical Engineering, Purdue University, West Lafayette, IN, United States; ^2^School of Mechanical Engineering, Purdue University, West Lafayette, IN, United States

**Keywords:** hemodynamic modeling, computational fluid dynamics, aneurysm, validation, fluid-structure interaction

## Abstract

Arterial aneurysms are pathological dilations of blood vessels, which can be of clinical concern due to thrombosis, dissection, or rupture. Aneurysms can form throughout the arterial system, including intracranial, thoracic, abdominal, visceral, peripheral, or coronary arteries. Currently, aneurysm diameter and expansion rates are the most commonly used metrics to assess rupture risk. Surgical or endovascular interventions are clinical treatment options, but are invasive and associated with risk for the patient. For aneurysms in locations where thrombosis is the primary concern, diameter is also used to determine the level of therapeutic anticoagulation, a treatment that increases the possibility of internal bleeding. Since simple diameter is often insufficient to reliably determine rupture and thrombosis risk, computational hemodynamic simulations are being developed to help assess when an intervention is warranted. Created from subject-specific data, computational models have the potential to be used to predict growth, dissection, rupture, and thrombus-formation risk based on hemodynamic parameters, including wall shear stress, oscillatory shear index, residence time, and anomalous blood flow patterns. Generally, endothelial damage and flow stagnation within aneurysms can lead to coagulation, inflammation, and the release of proteases, which alter extracellular matrix composition, increasing risk of rupture. In this review, we highlight recent work that investigates aneurysm geometry, model parameter assumptions, and other specific considerations that influence computational aneurysm simulations. By highlighting modeling validation and verification approaches, we hope to inspire future computational efforts aimed at improving our understanding of aneurysm pathology and treatment risk stratification.

## 1. Introduction

Arterial aneurysms are pathological focal dilations of arteries that can have life-threatening consequences. Aneurysms are commonly classified as saccular (asymmetric outpouchings) or fusiform (circumferential dilations). Other distinct vascular pathologies include pseudoaneurysms, which are partial thickness dilations of the blood vessel wall, and arterial dissections, which occur when medial layers separate and pressurized blood extravasates into a false lumen (Kumar et al., 2018). Aneurysm complications include rupture, hypovolemic shock (Dawson and Fitridge, 2013; Wanhainen et al., 2019), tissue compression (Thompson et al., 2015), dissection initiation or progression (Czerny et al., 2019), or thromboembolism and ischemia (Dawson and Fitridge, 2013; McCrindle et al., 2017). Aneurysm pathophysiology can involve endothelial changes, damage resulting in an inflammatory cascade, release of proteases, extracellular matrix remodeling, and smooth muscle cell apoptosis, all of which can propagate aneurysm growth and rupture (Chalouhi et al., 2012; Hendel et al., 2015) requiring surgical repair, coiling, and flow diverting stents (Table S1). Because there is abnormal blood flow and endothelial cell damage in aneurysms, coagulability can be pharmacologically altered to lower thrombus risk (McCrindle et al., 2017).

Current guidelines for aneurysm intervention consider diameter, expansion rate, symptoms, and other risk factors as summarized in Table S1. Since risk factors for aneurysm rupture prediction are imperfect considering that some small, growing aneurysms still rupture, it is likely that some large or rapidly growing aneurysms do not require surgical treatment (UCAS Japan Investigators et al., 2012; Dawson and Fitridge, 2013; Kontopodis et al., 2016; Saeyeldin et al., 2019). Similarly, the risk assessment of thromboembolism from aneurysms based on diameter has relatively poor sensitivity and specificity (Grande Gutierrez et al., 2019). However, assessing aneurysm hemodynamics with computational models may help identify more accurate predictors of vessel rupture or thrombosis formation, improving risk stratification that can guide clinical decision-making. This mini-review highlights recent literature that describes computational modeling of aneurysms and pseudoaneurysms using patient-specific geometries, boundary conditions, and model validation and verification.

## 2. From Images to Simulations

### 2.1. Imaging

Medical imaging can be used to acquire patient-specific information for computational fluid dynamics (CFD) and fluid-structure interaction (FSI) simulations. Vessel geometry information is often obtained using digital subtraction angiography (DSA), computed tomographic angiography (CTA), or magnetic resonance angiography (MRA). Recent efforts have also utilized volumetric ultrasound or optical coherence tomography to acquire vessel geometry (Jia et al., 2012; Van Disseldorp et al., 2019), but their application to CFD remains limited to animal studies (Phillips et al., 2017).

Every imaging technique has trade-offs. Most notably, DSA and CTA subject the patient to ionizing radiation, which can increase cancer incidence, limiting use for longitudinal studies (Einstein et al., 2007). Even so, the high resolution, low cost, and fast scan time have made CTA a common clinical imaging modality for aneurysms. While MRA does not subject patients to radiation, this technique requires expensive equipment, consists of longer scan times, and produces relatively low resolution images compared to CTA (Sailer et al., 2014).

In addition to aneurysm geometry, some imaging techniques provide subject-specific boundary conditions for hemodynamic simulations. Velocity information acquired non-invasively via pulsed wave Doppler ultrasound and phase contrast-magnetic resonance imaging (PC-MRI) can estimate two-dimensional velocity-based inlet boundary conditions (Boussel et al., 2009; Enevoldsen et al., 2012). PC-MRI provides time-resolved velocity measurements in either a single direction (2D PC-MRI) or throughout an entire volume (4D flow MRI) (Boussel et al., 2009; Eker et al., 2015), but is limited by lower spatio-temporal resolution. While each imaging modality has limitations, imaging data is key to provide the subject-specific information regarding aneurysm geometry and boundary conditions necessary for hemodynamic modeling.

### 2.2. Modeling

**Figure 1:**
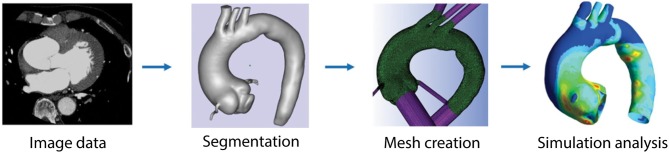
Pipeline used for computational modeling. Imaging data is acquired for the vessel of interest. The angiography images are segmented to identify the geometry of the vessel. Surface and volumetric meshes are created using available meshing software packages. Boundary conditions are defined and parameters are set in order to run simulations and analyze hemodynamic parameters, such as WSS, OSI, and others. Figure modified from Numata et al. (2016).

**Figure 2:**
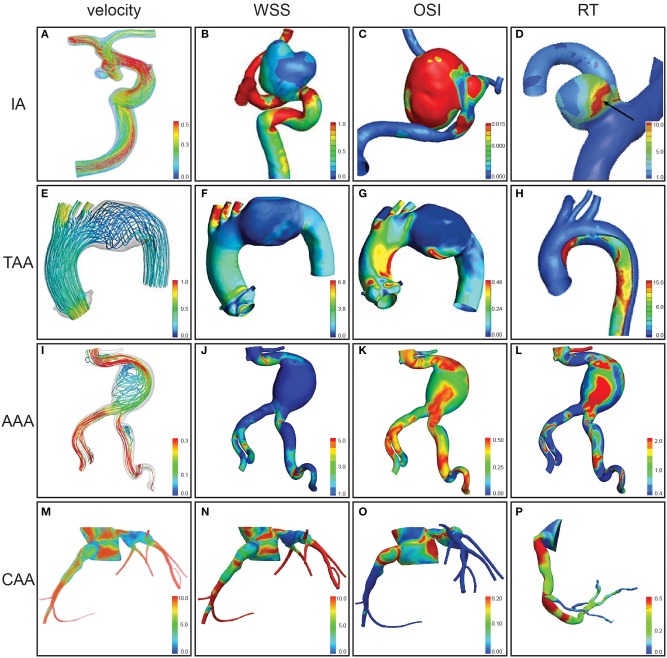
Hemodynamic parameters assessed by computational modeling for aneurysms at different anatomical locations. Velocity (m/s) **(A)**, velocity during peak systole (m/s) **(E,I)**, velocity during diastole (cm/s) **(M)**, wall shear stress (WSS) magnitude **(B)**, WSS in peak systole (Pa) **(F)**, WSS (Pa) **(J)**, WSS in diastole (dynes/sq cm) **(N)**, oscillatory shear index (OSI) **(C,G,K,O)**, relative residence time (RT) **(D,H,L)**, and particle RT gradient (s/m) **(P)** were assessed for intracranial aneurysms (IAs) of the internal carotid artery **(A)**, paraclinoid aneurysm in a segment of internal carotid artery **(B,C)**, and middle cerebral artery **(D)**, distal arch thoracic aortic aneurysm (TAA) **(E–G)**, thoracic aortic aneurysm dissection **(H)**, abdominal aortic aneurysm (AAA) **(I–L)**, and coronary artery aneurysms (CAAs) **(M–P)**. Figures modified from Tian et al. (2016) **(A)**, Wan et al. (2019) **(B–C)**, Sugiyama et al. (2013) **(D)**, Numata et al. (2016) **(E–G)**, Shi et al. (2016) **(H)**, Qiu et al. (2018) **(I–L)**, Sengupta et al. (2012), Sengupta (2013) **(M–O)**, and Sengupta et al. (2014) **(P)**.

Three-dimensional computational simulations of the vasculature can be used to estimate hemodynamic metrics. If the walls of the model are rigid, only the fluid domain is considered. If the walls are compliant, both solid and fluid domains are considered, often referred to as FSI simulations. The fluid domain model can be created by segmenting the vessel lumen from medical imaging data (Figure 1). The entire volume is then typically broken into discrete elements, creating a mesh of individual nodes (Figure 1, third panel). The conservation of mass and momentum equations for pressure and velocity can then be solved at each spatial location within the domain (Figure 2). This process varies depending on the study, software, and methods used. The most common parameters, inputs, as well as how they are obtained are listed in Table S2. Hemodynamic parameters that may influence aneurysm formation, growth, rupture, and thrombosis can be calculated based on the simulation results (Table S3). For example, wall shear stress (WSS) is the tangential stress blood exerts on vessel walls and has been linked to rupture risk (Figure 2B; Table S3; Meng et al., 2014). For risk stratification studies, WSS-related parameters have been investigated (Liang et al., 2019), such as WSS gradient (WSSG) (Table S3; Longo et al., 2017) and oscillatory shear index (OSI). OSI represents the change in direction of the shear forces during the cardiac cycle, and elevated OSI is associated with pro-inflammatory markers (Figure 2C; Sei et al., 2017). Complex flow patterns are frequently observed in aneurysms (Xiang et al., 2011). Flow stagnation in an aneurysm can be quantified by the residence time (RT), which is the average time a particle remains within the aneurysm (Reza and Arzani, 2019). Higher RT indicates flow stagnation and retention of platelets and inflammatory cells, which may contribute to thrombus formation (Figure 2D; Rayz et al., 2010; Reza and Arzani, 2019).

It is important to note that all models require assumptions, which can influence simulation results and model fidelity. This is especially important when estimating vessel wall properties, the inflow boundary conditions, and the outflow boundary conditions (Steinman and Pereira, 2019). While simulations are useful for calculating hemodynamic parameters linked to aneurysm progression, assumptions should be carefully considered to maintain reasonable model fidelity.

## 3. Aneurysms

### 3.1. Intracranial Aneurysm

Intracranial aneurysms (IAs) are present in 3.2% of the general population (Thompson et al., 2015). IAs are generally asymptomatic, but IA rupture can lead to subarachnoid hemorrhage (Thompson et al., 2015). While the management and risk stratification of IAs remains controversial (Cebral et al., 2011b) and there are no consensus guidelines with a diameter cut off (Thompson et al., 2015), a clinical standard for surgical intervention is defined as an aneurysm diameter ≥7–10 mm (Bederson et al., 2000; Thompson et al., 2015).

Patient-specific CFD simulations are now being used to identify hemodynamic parameters that may trigger IA growth or rupture (Etminan and Macdonald, 2015) (Figures 2A–D). Studies considering thrombosis often report RT, recirculation zones, and/or WSS as these are of interest for growth and rupture analyses (Vali et al., 2017). Initial conflicting results were reported for IA suggesting that aneurysm progression is related to both low WSS (Boussel et al., 2008; Miura et al., 2013) and high WSS (Cebral et al., 2009, 2011b). A unified theory by Meng et al. (2014) proposes two independent progression pathways. The first suggests that high WSS and a positive WSSG make a region prone to dilation, while the other suggests low WSS and high OSI are the driving forces. More recent work has correlated aneurysm growth and rupture to high OSI (Kawaguchi et al., 2012), high WSSG (Shojima et al., 2010; Machi et al., 2017), and larger areas of low WSS (Zhang et al., 2016; Qiu et al., 2017). Given that abnormally low WSS is related to endothelial cell damage, areas of low WSS may also correlate to areas of further vascular wall damage. Cebral et al. (2011b) identified additional qualitative risk factors within the aneurysmal region, including complex flow, unstable flow structures, concentrated inflows, and small impingement regions. Recent advancements in IA modeling have yielded the ability to aid in surgical planning and improve patient outcomes in several case studies (Vali et al., 2017). The ultimate aim to predict IA rupture risk on a patient-specific basis remains a work in progress (Saqr et al., 2019).

Unique challenges exist for modeling IAs. The small size of the cerebral vasculature makes accurate vessel segmentation and velocity measurements difficult. Although the cerebral vasculature is less elastic than proximal elastic arteries, a rigid wall assumption can alter WSS values and increase flow instability (Torii et al., 2007; Yamaguchi, 2016). Finally, a Newtonian blood flow assumption may be insufficient in some IA cases (Saqr et al., 2019). Similar to aneurysms in other parts of the body, relevant assumptions and approximate error bounds should be reported to improve reproducibility when modeling IAs.

### 3.2. Thoracic Aortic Aneurysms

Thoracic aortic aneurysms (TAAs) are pathological dilations of the thoracic aorta, most commonly occurring in the ascending region (Isselbacher, 2005; Ramanath et al., 2009). Hypertension, aging, and smoking all contribute to risk of aortic aneurysm development, and single gene mutations have greater influence on TAA development than any other region of the aorta (Milewicz et al., 2008; Hiratzka et al., 2009; Pinard et al., 2019). Current clinical guidelines suggest that rupture risk outweighs surgical risk for non-familial cases when TAA diameter is ≥55 mm or expands at a rate ≥5 mm/year (Hiratzka et al., 2009; Erbel et al., 2014). However, dissection and rupture occasionally occur below these thresholds, demonstrating a critical need for improved risk assessment (Pape et al., 2007; Zafar et al., 2018).

Since the ascending thoracic aorta sits directly above the left ventricle, it typically experiences the highest blood velocities, wall forces, and wall displacements of any artery. Computational modeling can be used to simulate these forces either using a rigid wall assumption or FSI methods to incorporate the effects of wall elasticity–a critical aspect in highly deformable vessels like the aorta (Reymond et al., 2013; Trachet et al., 2015) (Figures 2E–H). Studies using a rigid wall often focus on geometric effects using patient-specific parameters without the additional computational expense and required material properties needed for FSI. For example, studies using rigid walls revealed that patients with bicuspid aortic valve (BAV) have complex blood flow patterns that cause higher and uneven wall shear stresses, increasing the potential for TAA formation (Youssefi et al., 2017; Condemi et al., 2018; Edlin et al., 2019). Mendez et al. (2018) simulated blood flow in BAV TAAs using both rigid walls and FSI, finding non-significant differences in helical flow, but significantly lower estimated pressure in CFD simulations. In particular, they found the largest differences between rigid and deformable wall simulations occur during peak systole when wall deformation is greatest. Another study focusing on effects of hypertension and wall stiffness found that stiffer TAAs were correlated with the largest amount of altered wall stress distributions (Campobasso et al., 2018). Taken together, these computational studies demonstrate the importance of subject-specific modeling when simulating TAA initiation, progression, wall stresses, and rupture risk.

### 3.3. Abdominal Aortic Aneurysms

The abdominal aorta is particularly susceptible to aneurysm development and rupture, causing 175,000 deaths around the world each year (McGloughlin and Doyle, 2010; Howard et al., 2015). Similar to TAAs, abdominal aortic aneurysms (AAAs) ≥50–55 mm in diameter or have an expansion rate ≥10 mm in a year are recommended for surgical intervention (Erbel et al., 2014; Chaikof et al., 2018; Wanhainen et al., 2019).

Recent works suggest that simulations can be used to better predict rupture risk when incorporating WSS compared to a diameter only metric, as the interaction between AAA hemodynamics and vascular biomechanics has a stronger influence on eventual rupture (Taylor and Steinman, 2010; Canchi et al., 2015) (Figures 2I–L). A study using an *in vitro* model showed that AAA WSS was negatively influenced by intimal thickness (Bonert et al., 2003); the inverse correlation between WSS and wall thickness increases the risk of the rupture. Inclusion of an intraluminal thrombus, which most AAAs contain, and major neighboring branching vessels additionally produced more accurate flow simulations (Vorp, 2007; Salman et al., 2019).

Another computational study investigated blood flow characteristics at the site of AAA rupture and found that more patients experienced rupture in regions with low WSS (Boyd et al., 2016). This could be due to the observation that zones of recirculation are correlated with abundant thrombus deposition (Boyd et al., 2016). Velocity streamline patterns, which represent particle trajectories, tend to be stronger near the middle of the aorta (Soudah et al., 2013). While different shear stress levels can activate different methods of platelet deposition, several studies found that sites of low WSS and vortex formation suggested thrombus formation (Biasetti et al., 2010, 2011). Soudah et al. (2013) discovered a correlation between degree of AAA asymmetry and asymmetric flow pattern within the sac, which may lead to higher risk of endothelial dysfunction, thrombus formation, and eventual rupture. Overall, these studies illustrate how computational modeling has furthered our understanding of AAA progression and rupture, and future AAA modeling studies will provide even more knowledge about this disease.

### 3.4. Peripheral and Visceral Aneurysms

Peripheral artery aneurysms are located in the axillary, brachial, carotid, subclavian, femoral, and popliteal arteries, while visceral artery aneurysms are located in the splenic, celiac, superior and inferior mesenteric, renal, and hepatic arteries (Anderson et al., 2013; Dawson and Fitridge, 2013). These aneurysms are relatively rare with visceral aneurysms affecting 0.01–0.2% of the population (Huang et al., 2007). When detected, visceral aneurysms with a diameter ≥20 mm are typically treated to prevent rupture (Anderson et al., 2013). Peripheral aneurysms with a diameter ≥20–40 mm, depending on location, are treated to prevent thrombosis (Dawson and Fitridge, 2013). Idealized models of femoral artery pseudoaneurysms, where a saccular expansion develops often due to complications from percutaneous intervention, experience greater intraluminal pressures when the vessel to neck angle is larger (Suh et al., 2012). Further, simulations of common iliac artery aneurysms showed that as the size of the aneurysm increased, not only did WSS decrease, but remodeling of the upstream aorta also occurred (Parker et al., 2019). Finally, a study using pancreaticoduodenal artery aneurysm (PDAA) CFD models with superior mesenteric artery (SMA) occlusion resulted in analysis of the best treatment strategy. Surgery without revascularization could lead to recurrence of the PDAA, while revascularization could lead to high WSS and pressure (Li et al., 2019). These findings lead to stagnant flow in the pancreaticoduodenal artery (PDA), suggesting that revascularization can occlude the PDA and increase blood flow through the SMA (Li et al., 2019). Overall, the low prevalence, variation in anatomical location, and complex hemodynamics in peripheral and visceral vessels provide unique challenges when simulating blood flow. However, these lesions remain relatively under explored while still posing a clear risk to patients, highlighting the importance of studying hemodynamic changes via imaging and modeling to help improve clinical management.

### 3.5. Coronary Aneurysms

The incidence of coronary artery aneurysms (CAAs) is 1.65% (Abou Sherif et al., 2017). CAAs are found in patients with a history of Kawasaki disease (KD), a vasculitis, or atherosclerosis where the associated inflammatory processes weaken the vessel wall resulting in vessel dilation. CAAs can cause myocardial infarctions (MI) due to thrombosis (Abou Sherif et al., 2017). CAAs ≥8 mm in diameter or with a diameter z-score ≥10 are typically treated with anticoagulation therapy to prevent thrombus formation (McCrindle et al., 2017). Computational modeling of CAAs has the potential to better risk stratify these lesions and influence medical management (Figures 2M–P).

The first KD patient-specific model of a CAA was reported in Sengupta et al. (2012). To investigate the role of aneurysm hemodynamic parameters and morphology, KD patient coronary arteries (Sengupta et al., 2014) and CAAs with virtually simulated increased aneurysm length (fusiform shape) (Grande Gutierrez et al., 2017) were modeled. Grande Gutierrez et al. (2017) found that increasing aneurysm length increased the area exposed to low WSS, suggesting fusiform CAAs may elevate thrombosis risk relative to saccular CAAs while also demonstrating a limitation of using diameter for risk stratification. Recently, models from 10 KD patients retrospectively showed WSS-derived parameters and RT had improved specificity without loss in sensitivity compared to diameter-based metrics for thrombotic risk stratification (Grande Gutierrez et al., 2019). Models from 61 patients with atherosclerosis-caused CAAs demonstrated that CAAs with a length/diameter ratio >2 have elevated WSS derived parameters and increased incidence of MI (Fan et al., 2019). Together, these reports suggest that criteria based on diameter alone may be improved with additional simulation-based information.

While patient-specific computational modeling has the potential to better risk stratify coronary lesions, unique challenges exist when modeling CAAs. Specifically, the coronary arteries translate with cardiac motion and, unlike the rest of the systemic vasculature, diastolic blood flow is higher than systolic due to constriction of coronary vasculature when the heart contracts (Grande Gutierrez et al., 2019). Despite the use of diameter-based parameters for clinical risk stratification, fusiform aneurysms had non-significant increased risk of thrombosis compared to saccular CAAs (Sengupta et al., 2014). Future prospective studies with larger sample sizes could help determine the predictive clinical value of simulations when treating patients with these complex lesions.

## 4. Validation

Subject-specific computational model utility is limited by the degree to which models accurately represent reality. Modeling protocol and assumptions can influence model fidelity, which is assessed through verification, validation, and uncertainty quantification (VVUQ). Verification addresses the degree to which a model accurately solves the governing equations, while validation addresses the appropriateness of the equations and boundary conditions employed in the model. Uncertainty quantification addresses how numerical and physiological variations influence model results. Standards for VVUQ are well-established in the computational fluid dynamics community (Roache, 1994, 1997; Babuska and Oden, 2004), and several recent reviews have addressed the topic in the context of cardiovascular modeling (Taylor and Steinman, 2010; Steinman and Migliavacca, 2018; Steinman and Pereira, 2019).

VVUQ of aneurysm CFD models has recently gained greater attention as the field has matured (Cebral et al., 2011a; Fiorella et al., 2011; Putman et al., 2011; Steinman, 2011). Campobasso et al. (2018) modeled ascending TAAs, both verifying and validating their results with 4D flow MRI. However, since imaging methods for directly validating CFD model results *in vivo* are fraught with uncertainty themselves, direct validation remains challenging (Augst et al., 2003; Boussel et al., 2009). One approach to validate computational models is to compare results with experimental measurements *in vitro*; however, this does not validate the impact of assumptions used to create the computational model, including segmentation-induced geometrical errors and assumptions regarding vessel wall properties and boundary conditions. Validation of patient-specific cardiovascular modeling remains a challenge since both *in vivo* and *in vitro* approaches to measuring velocity fields have inherent limitations.

“Challenges” are an alternative to direct validation where several groups analyze a common data set and compare results (Berg et al., 2015; Janiga et al., 2015; Voß et al., 2019). Challenges can indicate the extent and impact of modeling assumption variability, while also suggesting ways to normalize to minimize variability in results. For example, Steinman et al. (2013) showed that differences in CFD solver settings on an IA model had relatively minor impact on peak systolic pressure drops (<8% variability) but significant impact on peak-systolic velocity entering the aneurysm sac. Valen-Sendstad et al. (2018) found large variability in sac-averaged WSS due to differences in segmentation, boundary conditions, and CFD solver settings that were reduced when normalizing by the parent vessel WSS. Voß et al. (2019) showed segmentation variability resulted in differences of 30% in mean aneurysmal velocity, 46% in neck inflow rate, and 51% in time-averaged WSS. Unlike most challenges, which do not have ground truth and therefore cannot assess accuracy, Berg et al. (2018) compared submitted geometries with higher resolution imaging data which served as the ground truth and validated that only one team correctly segmented the aneurysm necks. These reports suggest that multi-group, patient-specific modeling challenges can elucidate the impact of various modeling assumptions and suggest normalization approaches to better account for variability.

The degree to which computational models of arterial aneurysms have been validated varies widely with anatomical region; vascular territories where the field of computational modeling is more common and more mature tend to have more validation studies. For example, all of the modeling challenges mentioned were for IAs (Steinman et al., 2013; Berg et al., 2015, 2018, 2019; Janiga et al., 2015; Valen-Sendstad et al., 2018; Voß et al., 2019). Validation studies have also been performed for TAAs (e.g., Campobasso et al., 2018), AAAs (e.g., Kung et al., 2011), and CAAs (e.g., Kung et al., 2014), while the majority of modeling studies for PAAs and VAAs are case studies and generally do not include validation.

## 5. Outlook

Although there is tremendous hope for the use of CFD modeling of aneurysms to aid in clinical decision making, there are only a few instances of CFD in surgical planning or large clinical cohort studies for aneurysms. The majority of studies have focused on predicting hemodynamics and not rupture risk stratification, especially for less common aneurysm locations, including PAA and VAA (Table S1). The conversion of hemodynamic values into clinically applicable parameters has been studied for IAs (Xiang et al., 2011; Detmer et al., 2018a,b), but most TAAs, AAAs, PAAs, VAAs, and CAAs studies are exploratory (Table S1). For CFD modeling of an aneurysm to be used widely in the clinic, large scale multi-centered studies and the identification of standardized parameters or groups of parameters that predict risk with high sensitivity and specificity are typically required (Kallmes, 2012). Additionally, the infrastructure to input patient-specific data and output a clinically relevant parameter also needs to be developed. As clinicians do not typically have time in their schedules to build CFD models and run simulations themselves (Singh et al., 2009), alternative approaches could involve the commercialization of a modeling service as done by HeartFlow® to identify patients with coronary artery disease that require treatment (Danad et al., 2017; Patel et al., 2020). Beyond coronary disease, VASCOPS is a company that took a solid mechanics approach and developed A4clinics™ software using finite element modeling of the vessel wall to predict AAA rupture using Peak Wall Rupture Risk (VASCOPS, 2018). Its acceptance in the AAA field, however, has been limited as questions remain about clinical benefit (Chung et al., 2017; Boyd, 2020). For the widespread implementation of CFD modeling of aneurysms for clinical decision making, an artificial intelligence approach may be required followed by manual segmentation correction in order to handle a large number of cases (Taylor et al., 2018; Maher et al., 2019).

## 6. Conclusion

Computational modeling of the arterial systems is proving to be a valuable tool to study aneurysm growth, rupture, and thrombosis. Models can calculate hemodynamic parameters from complex geometries and have the potential to improve clinical decision-making. Common challenges include the incorporation of subject-specific boundary conditions, geometry, and error assessment due to modeling assumptions. Unique challenges exist in different anatomical locations, including vessel wall motion in TAAs and AAAs, flow during diastole in CAAs, anatomical variability in VAAs and PAAs, and anastomosis in IAs. The maturity of the field differs depending on location, from regions studied in depth, such as IAs and AAAs to more nascent areas, such as PAAs and VAAs (Table S1). Since predictive models are limited by the fidelity of the modeling assumptions, VVUQ efforts provide critical assessment of the accuracy of the model. As this field advances, more studies in diverse locations, use of large retrospective and prospective studies and longitudinal animal studies, and advancing from exploratory research to confirmatory studies could help fully capture the predictive value and clinical impact of computational hemodynamic modeling.

## Author Contributions

SL, EN, HC, TD, JM, SR, and KS wrote the sections of the manuscript. CG provided the initial guidance and edited all sections. All authors contributed to the manuscript revision and approved the submitted version.

## Conflict of Interest

The authors declare that the research was conducted in the absence of any commercial or financial relationships that could be construed as a potential conflict of interest.

## Abbreviations

AAAs: abdominal aortic aneurysms
BAV: bicuspid aortic valve
CAAs: coronary artery aneurysms
CFD: computational fluid dynamics
CTA: computed tomographic angiography
DSA: digital subtraction angiography
FSI: fluid-structure interactions
IAs: intracranial aneurysms
KD: Kawasaki disease
MI: myocardial infarct
MRA: magnetic resonance angiography
MRI: magnetic resonance imaging
OSI: oscillatory shear index
PAAs: peripheral artery aneurysms
PDA: pancreaticoduodenal artery
PDAA: pancreaticoduodenal artery aneurysm
PC-MRI: phase contrast-magnetic resonance imaging
SMA: superior mesenteric artery
RT: residence time
TAAs: thoracic aortic aneurysms
WSS: wall shear stress
WSSG: wall shear stress gradient
VAAs: visceral artery aneurysms
VVUQ: verification, validation, and uncertainty quantification.
