# Supplementary material for: Computational Hemodynamic Modeling of Arterial Aneurysms: A Mini-Review
Source: Front Physiol. 2020 May 12;11:454. doi: 10.3389/fphys.2020.00454 (PMC7235429; doi:10.3389/fphys.2020.00454)
Supplement: Supplementary file 1 [file Table_1.pdf]

Supplemental material for *Lipp SN, Niedert EE, Cebull HL, Diorio TC, Ma JL, Rothenberger SM, Stevens Boster KA and Goergen CJ (2020) Computational Hemodynamic Modeling of Arterial Aneurysms: A Mini-Review. Front. Physiol. 11:454. doi: 10.3389/fphys.2020.00454*

Table S1: Comparison of aneurysm epidemiology, morphology, clinical treatments, intervention criteria, simulation considerations, and types of clinical studies. Abbreviations: D, Diameter; KD, Kawasaki disease

location	epidemiology and morphology	intervention criteria and current treatments	simulation considerations and types of clinical studies
**all locations**	connective tissue disorder, atherosclerosis [1–10]	diameter, diameter-based z-score, or rate of increasing diameter[2–4,11–14]	subject-specific boundary conditions and geometry are needed[15]
**cerebral aneurysms (IAs)**	**risk factors**: hypertension, smoking, atherosclerosis, family history of subarachnoid hemorrhage, polycystic kidney disease, connective tissue disorders, and female sex[1,16]<br>**prevalence**: 3.2% (95% confidence interval 1.9% - 5.2%[1])<br>**morbidity/mortality**: 60% mortality rate within the first 6 months of a subarachnoid hemorrhage[16]<br>**morphology**: saccular (90%, 85% of which are at the Circle of Willis), fusiform, dissecting, and micotic[17]	**concern**: subarachnoid hemorrhage[1]<br>**treatment**: endovascular coiling, neurosurgical clipping, and endovascular stents[1]<br>**surgical intervention**: based on clinical history, family history, location, and size (D ≥ 7 - 10 mm)[1,11,16,18]; however, the most recent consensus guidelines do not define a diameter cutoff[1]	• proper location of vessel truncation[19] and inclusion of side arterial branches[20] improves accuracy<br>• small vessel size reduces Newtonian blood flow assumption[21], segmentation[22], and velocity measurement[23] accuracy<br>• small aneurysm size limits boundary condition and geometry sources (reduced accuracy with 4D flow MRI[24]; limited use with ultrasound)<br>• rigid wall assumption can alter WSS values and increase flow instability[25,26]<br>➢ **case series surgical planning**[27,28], **studies comparing rupture cases to unruptured controls**[29,30], **large studies comparing rupture cases to control**[31–34], **prospective study**[35]
**thoracic aortic aneurysms (TAAs)**	**risk factors**: connective tissue disorder (e.g. Marfan syndrome), aortic valvular disease (bicuspid valve), hypertension, smoking, and infection (syphilis)[2,6]<br>**prevalence**: 0.16 - 0.34%[36,37]	**concern**: dissection, rupture[2]<br>**treatment**: open aneurysm repair or endovascular aneurysm repair[2]<br>**surgical intervention**: D ≥ 55 mm[2,40] or a growth rate of ≥ 5	• pulsatile helical flow observed[41,42]<br>• deformable walls can account for the influence of vessel wall motion on hemodynamics[43]

location	epidemiology and morphology	intervention criteria and current treatments	simulation considerations and types of clinical studies
	**morbidity/mortality**: acute dissection 27.4% and rupture is fatal for 59% before reaching the hospital[38]<br>**morphology**: fusiform > saccular[6], the majority occur in the root or ascending aorta (~60%)[39]	mm/year with considerations for genetic syndromes or bicuspid valve and other factors[2,6]	• dissection or thrombus may alter geometries [44,45]<br>➢ **case study[45], case compared to control[46], treatment options[47–49]**
**abdominal aortic aneurysms (AAAs)**	**risk factors**: smoking, connective tissue disorder, atherosclerosis, hypertension, and male sex (but rupture is associated with female sex)[2–4]<br>**prevalence**: 1.4%[4]<br>**morbidity/mortality**: > 80% mortality with rupture[3]<br>**morphology**: fusiform more common[3]	**concern**: rupture[3]<br>**treatment**: open aneurysm repair, endovascular aneurysm repair[3]<br>**surgical intervention**: D ≥ 50 - 55 mm or 10 mm/year[2,3,4]	• pulsatile simulations capture transient formation and disappearance of vortices[50–53]<br>• inclusion of material properties and aortic dynamics can mimic vessel wall behavior[51], but rigid wall sufficiently quantifies blood flow dynamics[54]<br>• can couple with growth and remodeling studies to assess AAAs dynamics[51]<br>• inclusion of an intraluminal thrombus and major neighboring branching vessels improves accuracy[51,55,56]<br>➢ **case study[57] and treatment options[58–61]**
**peripheral artery aneurysms (PAAs)**	**carotid, axillary, brachial, femoral, and popliteal arteries**<br>**risk factors**: hypertension, atherosclerosis[9]<br>smoking[10], trauma (femoral artery pseudoaneurysm), and male sex is associated with iliac, femoral, and popliteal artery aneurysms[62–64]<br>**prevalence**: not well known and varies across locations (popliteal artery aneurysm are found in 1% men over 65)[12,62]	**concern**: thrombosis and rupture[12]<br>**treatment**: open aneurysm repair, or endovascular aneurysm repair[12]<br>**surgical intervention**: if D ≥ 20-40 mm, and depends on location, reviewed in[12,13]	• low prevalence[12,62] limits data sets[65]<br>• variation in anatomical location[65] complicates models<br>➢ **case study in an idealized geometry[66], comparisons between ruptured and unruptured aneurysm[65]**

location	epidemiology and morphology	intervention criteria and current treatments	simulation considerations and types of clinical studies
	**morbidity/mortality**: thromboembolic complications for popliteal artery aneurysm 35% (8 - 100%)[9]<br>**morphology**: saccular, fusiform[9]		
**visceral artery aneurysms (VAAs)**	**splenic, celiac, superior and inferior mesenteric, renal, and hepatic arteries**<br>**risk factor**: connective tissue disorders, atherosclerosis, fibromuscular dysplasia, trauma, vasculitis, hypertension, pregnancy (splenic), splenic and renal artery aneurysm associated with female sex, and hepatic artery aneurysm associated with male sex[7,8]<br>**incidence**: 1% (range 0.098% - 10.4%)[8] 0.01 - 0.2%[67]<br>**morbidity/mortality**: 25 - 70% mortality rate depending on location[8,67]<br>**morphology**: saccular or fusiform depends on location[8]	**concern**: rupture[12]<br>**treatment**: open aneurysm surgical repair or endovascular aneurysm repair[13]<br>**surgical intervention**: if D ≥ 20 mm[12,13]	• low prevalence[8,67] limits data sets<br>• variation in geometries and anatomical location of the aneurysm[68] complicate models<br>➢ **case study**[69], **case study for surgical planning**[70]
**coronary artery aneurysms (CAAs)**	**risk factors**: Kawasaki disease (KD) and other vasculitides, atherosclerosis, trauma, connective tissue disorders, and male sex[5]<br>**incidence**: 1.65% (range 0.3 - 5.3%)[5]<br>**morbidity/mortality**: 5% of patients with myocardial infarct less than 40 years old have CAA with a history of KD[71]<br>**morphology**: complex in shape, saccular, fusiform, or appear as a string of pearls[72,73]	**concern**: thrombosis[14]<br>**treatment**: antiplatelet and anticoagulation therapies[14]<br>**medical intervention**: antiplatelet therapies (z-score ≥ 2) and antiplatelet therapies and anticoagulation treatment if D ≥ 8 mm or a z-score ≥ 10[14]	• coronary arteries translate with cardiac motion[74]<br>• blood flow occurs during diastole[74]<br>➢ **case studies and series**[74,75], **cases compared to controls without aneurysm**[73] **or without sequala**[76,77]

Table S2: Modeling parameters and inputs commonly used in computational modeling of aneurysms[51]

common modeling parameters	common inputs	how commonly obtained
arterial geometry	3D model created from segmentation of medical images; common segmentation software[51] includes MIMICS, VMTK[78], ITK-SNAP, etc.	magnetic resonance imaging, computed tomography, ultrasound, etc.
numerical settings	software/numerical scheme dependent; common solvers include SimVascular[79], Crimson, ANSYS Fluent[80,81], ANSYS CFX[82], STAR CCM+[83], OpenFOAM[84], Oasis, FEBio[85], etc.	based on experience, from literature, derived during verification studies
inlet boundary condition(s)	flow waveform and profile[86,87]	literature, *in vivo* flow measurements[78], eg. phase contrast magnetic resonance imaging, catheter-based probes, ultrasound
outlet boundary condition(s)	pressure[51,86,87]	literature, *in vivo* flow measurements, eg. catheter-based probes[51]
	lumped parameter models[86,87] (e.g. Windkessel)	literature, morphometric relations (e.g. generalized Murray's law), *in vivo* flow measurements, iterative tuning
FSI material properties	wall thickness[51,88,89,90], elasticity[51]	literature, histology from resected tissue for wall thickness, biomechanical tests from resected tissue for elasticity
fluid properties	Newtonian, incompressibility, density, viscosity[91]	literature

Table S3: Hemodynamic parameters commonly used in computational modeling of aneurysm[92]

hemodynamic parameter	nomenclature/equation	common units	hemodynamic considerations
**wall shear stress (WSS)**	$\overrightarrow{\tau}_{w} = \mu \frac{\partial v}{\partial r}$, evaluated at the wall	Dynes/cm^2, Pa	measure of hemodynamic stress on vessel wall - related to thrombosis risk and endothelial function; low levels associated with proinflammatory and prothrombotic and high levels associated with vascular disease pathogenesis[93,94]
**time-averaged wall shear stress (TAWSS)**	$TAWSS = \frac{1}{t}\int_0^t \lvert WSS \rvert \, dt$	Dynes/cm^2, Pa	
**wall shear stress gradient (WSSG)**	$WSSG = \sqrt{\left(\left\lvert\frac{\partial \vec{\tau}_w}{\partial x}\right\rvert\right)^2 + \left(\left\lvert\frac{\partial \vec{\tau}_w}{\partial y}\right\rvert\right)^2 + \left(\left\lvert\frac{\partial \vec{\tau}_w}{\partial z}\right\rvert\right)^2}$	Dynes/cm^3, Pa/mm	magnitude of spatial gradient of WSS, positive WSSG (i.e. accelerating flow) associated with cerebral aneurysm growth and remodeling[93]
**oscillatory shear index (OSI)**	$OSI = 0.5\left(1 - \frac{\lvert \int_0^t \overrightarrow{WSS} dt \rvert}{\int_0^t \lvert \overrightarrow{WSS} \rvert dt}\right)$	dimensionless	measure of flow directionality and disturbed flow, associated with proinflammatory changes[93] minimum OSI = 0: unidirectional flow; maximum OSI = 0.5: equal flow in both directions[92]
**residence time (RT) measures**	quantifiable via Lagrangian or Eulerian methods[95]	s, 1/Pa, dimensionless	measure of flow stagnation, related to thrombosis and wall inflammation[95,96]<br><br>there is no globally appropriate RT method; approach should be selected based on the context of the simulation and quantities of interest[95]

References

1. Thompson, B. G. *et al.* Guidelines for the management of patients with unruptured intracranial aneurysms: a guideline for healthcare professionals from the American Heart Association/American Stroke Association. *Stroke.* **46**, 2368–2400 (2015).
2. Erbel, R. *et al.* 2014 ESC guidelines on the diagnosis and treatment of aortic diseases. *Eur. Heart J.* **35**, 2873–2926 (2014).
3. Wanhainen, A. *et al.* Editor's Choice – European Society for Vascular Surgery (ESVS) 2019 clinical practice guidelines on the management of abdominal aorto-iliac artery aneurysms. *Eur. J. Vasc. Endovasc. Surg.* **57**, 8–93 (2019).
4. Chaikof, E. L. *et al.* The Society for Vascular Surgery practice guidelines on the care of patients with an abdominal aortic aneurysm. *J. Vasc. Surg.* **67**, 2–77.e2 (2018).
5. Abou Sherif, S., Ozden Tok, O., Taskoylu, O., Goktekin, O. & Kilic, I. D. Coronary artery aneurysms: a review of the epidemiology, pathophysiology, diagnosis, and treatment. *Front Cardiovasc Med* **4**, 24 (2017).
6. Hiratzka, L. F. *et al.* 2010 ACCF/AHA/AATS/ACR/ASA/SCA/SCAI/SIR/STS/SVM Guidelines for the diagnosis and management of patients with thoracic aortic disease. *Circulation* **121**, 266–369 (2010).
7. Nosher, J. L., Chung, J., Brevetti, L. S., Graham, A. M. & Siegel, R. L. Visceral and renal artery aneurysms: a pictorial essay on endovascular therapy. *Radiographics* **26**, 1687–1704 (2006).
8. Panayiotopoulos, Y. P., Assadourian, R. & Taylor, P. R. Aneurysms of the visceral and renal arteries. *Ann. R. Coll. Surg. Engl.* **78**, 412–9 (1996).
9. Dawson, I., Sie, R. B. & van Bockel, J. H. Atherosclerotic popliteal aneurysm. *Br. J. Surg.* **84**, 293–299 (1997).
10. Martelli, E. *et al.* Popliteal artery aneurysms. Factors associated with thromboembolism and graft failure. *Int. Angiol.* **23**, 54–65 (2004).
11. Matsubara, S., Hadeishi, H., Suzuki, A., Yasui, N. & Nishimura, H. Incidence and risk factors for the growth of unruptured cerebral aneurysms: observation using serial computerized tomography angiography. *J. Neurosurg.* **101**, 908–914 (2004).
12. Dawson, J. & Fitridge, R. Update on aneurysm disease: current insights and controversies: peripheral aneurysms: when to intervene – is rupture really a danger? *Prog. Cardiovasc. Dis.* **56**, 26–35 (2013).
13. Anderson, J. L. *et al.* Management of patients with peripheral artery disease (compilation of 2005 and 2011 ACCF/AHA guideline recommendations): a report of the American College of Cardiology Foundation/American Heart Association task force on practice guidelines. *J. Am. Coll. Cardiol.* **61**, 1555–1570 (2013).
14. McCrindle, B. W. *et al.* Diagnosis, treatment, and long-term management of Kawasaki Disease: a scientific statement for health professionals from the American Heart Association. *Circulation* **135**, e927–e999 (2017).
15. Jansen, I. G. H. *et al.* Generalized versus patient-specific inflow boundary conditions in computational fluid dynamics simulations of cerebral

aneurysmal hemodynamics. *Am. J. Neuroradiol.* **35**, 1543–1548 (2014).

16. Steiner, T. *et al.* European Stroke Organization guidelines for the management of intracranial aneurysms and subarachnoid haemorrhage general considerations. *Cerebrovasc. Dis.* **35**, 93–112 (2013).

17. Gasparotti, R. & Liserre, R. Intracranial aneurysms. *Eur. Radiol.* **15**, 441–447 (2005).

18. Bederson, J. B. *et al.* Recommendations for the management of patients with unruptured intracranial aneurysms: a statement for healthcare professionals from the Stroke Council of the American Heart Association. *Stroke* **31**, 2742–2750 (2000).

19. Pereira, V. M. *et al.* Evaluation of the influence of inlet boundary conditions on computational fluid dynamics for intracranial aneurysms: a virtual experiment. *J. Biomech.* **46**, 1531–1539 (2013).

20. Krylov, V., Grigoryeva, E., Dolotova, D., Blagosklonova, E. & Gavrilov, A. CFD modelling of local hemodynamics in intracranial aneurysms harboring arterial branches. *Stud. Health Technol. Inform.* **238**, 64–67 (2017).

21. Saqr, K. M. *et al.* What does computational fluid dynamics tell us about intracranial aneurysms? A meta-analysis and critical review. *J. Cereb. Blood Flow Metab.* **40**, 1021–1039 (2020).

22. Berg, P. *et al.* 3DRA reconstruction of intracranial aneurysms - how does voxel size influences morphologic and hemodynamic parameters. *Proc. Annu. Int. Conf. IEEE Eng. Med. Biol. Soc.* **2018–July**, 1327–1330 (2018).

23. Dyverfeldt, P. *et al.* 4D flow cardiovascular magnetic resonance consensus statement. *J. Cardiovasc. Magn. Reson.* **17**, 1–19 (2015).

24. Itatani, K. *et al.* New imaging tools in cardiovascular medicine: computational fluid dynamics and 4D flow MRI. *Gen. Thorac. Cardiovasc. Surg.* **65**, 611–621 (2017).

25. Yamaguchi, R. Effect of elasticity on flow characteristics inside intracranial aneurysms. *Int. J. Neurol. Neurother.* **3**, 049 (2016).

26. Torii, R., Oshima, M., Kobayashi, T., Takagi, K. & Tezduyar, T. E. Influence of wall elasticity in patient-specific hemodynamic simulations. *Comput. Fluids* **36**, 160–168 (2007).

27. Vali, A., Abla, A. A., Lawton, M. T., Saloner, D. & Rayz, V. L. Computational fluid dynamics modeling of contrast transport in basilar aneurysms following flow-altering surgeries. *J. Biomech.* **50**, 195–201 (2017).

28. Rayz, V. L. *et al.* Computational modeling of flow-altering surgeries in basilar aneurysms. *Ann. Biomed. Eng.* **43**, 1210–1222 (2015).

29. Pereira, V. M. *et al.* Wall shear stress distribution of small aneurysms prone to rupture: a case-control study. *Stroke* **45**, 261–4 (2014).

30. Liu, J., Fan, J., Xiang, J., Zhang, Y. & Yang, X. Hemodynamic characteristics of large unruptured internal carotid artery aneurysms prior to rupture: a case control study. *J. Neurointerv. Surg.* **8**, 367–372 (2016).

31. Detmer, F. J. *et al.* Development and internal validation of an aneurysm rupture probability model based on patient characteristics and

aneurysm location, morphology, and hemodynamics. *Int. J. Comput. Assist. Radiol. Surg.* **13**, 1767–1779 (2018).

32. Detmer, F. J. *et al.* External validation of cerebral aneurysm rupture probability model with data from two patient cohorts. *Acta Neurochir. (Wien).* **160**, 2425–2434 (2018).

33. Xiang, J. *et al.* Hemodynamic-morphologic discriminants for intracranial aneurysm rupture. *Stroke* **42**, 144–152 (2011).

34. Xiang, J. *et al.* Rupture resemblance score (RRS): toward risk stratification of unruptured intracranial aneurysms using hemodynamic-morphological discriminants. *J. Neurointerv. Surg.* **7**, 490–495 (2015).

35. Talari, S. *et al.* Comparison of computational fluid dynamics findings with intraoperative microscopy findings in unruptured intracranial aneurysms- an initial analysis. *Asian J. Neurosurg.* **11**, 356 (2016).

36. Itani, Y. *et al.* Measurement of aortic diameters and detection of asymptomatic aortic aneurysms in a mass screening program using a mobile helical computed tomography unit. *Heart Vessels* **16**, 42–45 (2002).

37. Kälsch, H. *et al.* Body-surface adjusted aortic reference diameters for improved identification of patients with thoracic aortic aneurysms: results from the population-based Heinz Nixdorf Recall study. *Int. J. Cardiol.* **163**, 72–78 (2013).

38. Kuzmik, G. A., Sang, A. X. & Elefteriades, J. A. Natural history of thoracic aortic aneurysms. *J. Vasc. Surg.* **56**, 565–571 (2012).

39. Isselbacher, E. M. Thoracic and abdominal aortic aneurysms. *Circulation* **111**, 816–828 (2005).

40. Czerny, M. *et al.* Current options and recommendations for the treatment of thoracic aortic pathologies involving the aortic arch. *Eur. J. Cardio-Thoracic Surg.* **55**, 133–162 (2019).

41. Youssefi, P. *et al.* Impact of patient-specific inflow velocity profile on hemodynamics of the thoracic aorta. *J. Biomech. Eng.* **140**, 011002 (2018).

42. Youssefi, P., Sharma, R., Figueroa, C. A. & Jahangiri, M. Functional assessment of thoracic aortic aneurysms - the future of risk prediction? *Br. Med. Bull.* **121**, 61–71 (2017).

43. Mendez, V., Di Giuseppe, M. & Pasta, S. Comparison of hemodynamic and structural indices of ascending thoracic aortic aneurysm as predicted by 2-way FSI, CFD rigid wall simulation and patient-specific displacement-based FEA. *Comput. Biol. Med.* **100**, 221–229 (2018).

44. Shi, Y., Zhu, M., Chang, Y., Qiao, H. & Liu, Y. The risk of stanford type-A aortic dissection with different tear size and location: a numerical study. *Biomed. Eng. Online* **15**, 128 (2016).

45. Borghi, A., Wood, N. B., Mohiaddin, R. H. & Xu, X. Y. 3D geometric reconstruction of thoracic aortic aneurysms. *Biomed. Eng. Online* **5**, (2006).

46. Numata, S. *et al.* Blood flow analysis of the aortic arch using computational fluid dynamics. *Eur. J. Cardio-Thoracic Surg.* **49**, 1578–1585 (2016).

47. Menichini, C. *et al.* High wall stress may predict the formation of stent-graft–induced new entries after thoracic endovascular aortic repair. *J. Endovasc. Ther.* **25**, 571–577 (2018).

48. Ma, T. *et al.* Computational investigation of interaction between stent graft and aorta in retrograde type A dissection after thoracic endovascular aortic repair for type B aortic dissection. *J. Vasc. Surg.* **68**, 14S–21S.e2 (2018).

49. Romarowski, R. M. *et al.* Computational simulation of TEVAR in the ascending aorta for optimal endograft selection: a patient-specific case study. *Comput. Biol. Med.* **103**, 140–147 (2018).

50. Finol, E. A. & Amon, C. H. Flow-induced wall shear stress in abdominal aortic aneurysms: part II - pulsatile flow hemodynamics. *Comput. Methods Biomech. Biomed. Engin.* **5**, 319–328 (2002).

51. Salman, H. E., Ramazanli, B., Yavuz, M. M. & Yalcin, H. C. Biomechanical investigation of disturbed hemodynamics-induced tissue degeneration in abdominal aortic aneurysms using computational and experimental techniques. *Front. Bioeng. Biotechnol.* **7**, (2019).

52. Yu, S. C. M. & Zhao, J. B. A particle image velocimetry study on the pulsatile flow characteristics in straight tubes with an asymmetric bulge. *Proc. Inst. Mech. Eng. Part C J. Mech. Eng. Sci.* **214**, 655–671 (2000).

53. Stamatopoulos, C., Mathioulakis, D. S., Papaharilaou, Y. & Katsamouris, A. Experimental unsteady flow study in a patient-specific abdominal aortic aneurysm model. *Exp. Fluids* **50**, 1695–1709 (2011).

54. Lin, S., Han, X., Bi, Y., Ju, S. & Gu, L. Fluid-structure interaction in abdominal aortic aneurysm: effect of modeling techniques. *Biomed Res. Int.* **2017**, (2017).

55. Vorp, D. A. Biomechanics of abdominal aortic aneurysm. *J. Biomech.* **40**, 1887–902 (2007).

56. Gasser, T. C., Auer, M., Labruto, F., Swedenborg, J. & Roy, J. Biomechanical rupture risk assessment of abdominal aortic aneurysms: model complexity versus predictability of finite element simulations. *Eur. J. Vasc. Endovasc. Surg.* **40**, 176–185 (2010).

57. Qiu, Y., Yuan, D., Wang, Y., Wen, J. & Zheng, T. Hemodynamic investigation of a patient-specific abdominal aortic aneurysm with iliac artery tortuosity. *Comput. Methods Biomech. Biomed. Engin.* **21**, 824–833 (2018).

58. Raptis, A. *et al.* Hemodynamic profile of two aortic endografts accounting for their postimplantation position. *J. Med. Device.* **11**, (2017).

59. Kandail, H., Hamady, M. & Xu, X. Y. Comparison of blood flow in branched and fenestrated stent-grafts for endovascular repair of abdominal aortic aneurysms. *J. Endovasc. Ther.* **22**, 578–590 (2015).

60. Casciaro, M. E. *et al.* Effects on aortoiliac fluid dynamics after endovascular sealing of abdominal aneurysms. *Vasc. Endovascular Surg.* **52**, 621–628 (2018).

61. Sughimoto, K. *et al.* Blood flow dynamic improvement with aneurysm repair detected by a patient-specific model of multiple aortic aneurysms. *Heart Vessels* **29**, 404–412 (2014).

62. Lawrence, P. F., Lorenzo-Rivero, S. & Lyon, J. L. The incidence of iliac, femoral, and popliteal artery aneurysms in hospitalized patients. *J. Vasc. Surg.* **22**, 409–416 (1995).

63. Stone, P. A., Campbell, J. E. & AbuRahma, A. F. Femoral pseudoaneurysms after percutaneous access. *J. Vasc. Surg.* **60**, 1359–1366 (2014).

64. Hall, H. A., Minc, S. & Babrowski, T. Peripheral artery aneurysm. *Surg. Clin. North Am.* **93**, 911–923 (2013).

65. Parker, L. P. *et al.* Morphology and hemodynamics in isolated common iliac artery aneurysms impacts proximal aortic remodeling. *Arterioscler. Thromb. Vasc. Biol.* **39**, 1125–1136 (2019).

66. Suh, S.-H., Kim, H.-H., Choi, Y. H. & Lee, J. S. Computational fluid dynamic modeling of femoral artery pseudoaneurysm. *J. Mech. Sci. Technol.* **26**, 3865–3872 (2012).

67. Huang, Y. K. *et al.* Visceral artery aneurysm: risk factor analysis and therapeutic opinion. *Eur. J. Vasc. Endovasc. Surg.* **33**, 293–301 (2007).

68. Mazzaccaro, D., Malacrida, G. & Nano, G. Variability of origin of splanchnic and renal vessels from the thoracoabdominal aorta. *Eur. J. Vasc. Endovasc. Surg.* **49**, 33–38 (2015).

69. Iimuro, Y. *et al.* Hemodynamic analysis and treatment of an enlarging extrahepatic portal aneurysm: report of a case. *Surg. Today* **45**, 383–389 (2015).

70. Li, D. *et al.* Hemodynamic analysis to assist treatment strategies in complex visceral aterial pathologies: case reports and discussion from pancreaticoduodenal artery aneurysm with superior mesenteric artery occlusion. *Ann. Vasc. Surg.* **59**, 308.e1-308.e8 (2019).

71. Daniels, L. B. *et al.* Prevalence of Kawasaki disease in young adults with suspected myocardial ischemia. *Circulation* **125**, 2447–53 (2012).

72. Onouchi, Z., Shimazu, S., Kiyosawa, N., Takamatsu, T. & Hamaoka, K. Aneurysms of the coronary arteries in Kawasaki disease. An angiographic study of 30 cases. *Circulation* **66**, 6–13 (1982).

73. Sengupta, D. *et al.* Thrombotic risk stratification using computational modeling in patients with coronary artery aneurysms following Kawasaki disease. *Biomech. Model. Mechanobiol.* **13**, 1261–1276 (2014).

74. Sengupta, D. *et al.* Image-based modeling of hemodynamics in coronary artery aneurysms caused by Kawasaki disease. *Biomech. Model. Mechanobiol.* **11**, 915–932 (2012).

75. Grande Gutierrez, N., Kahn, A., Burns, J. C. & Marsden, A. L. Computational blood flow simulations in Kawasaki disease patients: insight into coronary artery aneurysm hemodynamics. *Glob. Cardiol. Sci. Pract.* **2017**, e201729 (2017).

76. Grande Gutierrez, N. *et al.* Hemodynamic variables in aneurysms are associated with thrombotic risk in children with Kawasaki disease. *Int. J. Cardiol.* **281**, 15–21 (2019).

77. Fan, T. *et al.* Morphometry and hemodynamics of coronary artery aneurysms caused by atherosclerosis. *Atherosclerosis* **284**, 187–193 (2019).

78. McGah, P. M. *et al.* Accuracy of computational cerebral aneurysm hemodynamics using patient-specific endovascular measurements. *Ann. Biomed. Eng.* **42**, 503–514 (2014).

79. Updegrove, A. *et al.* SimVascular: An Open Source Pipeline for Cardiovascular Simulation. *Annals of Biomedical Engineering* **45**, 525–541 (2017).

80. Botti, L., Paliwal, N., Conti, P., Antiga, L. & Meng, H. Modeling hemodynamics in intracranial aneurysms: Comparing accuracy of CFD solvers based on finite element and finite volume schemes. *Int. j. numer. method. biomed. eng.* **34**, e3111 (2018).

81. Jain, K., Jiang, J., Strother, C. & Mardal, K.-A. Transitional hemodynamics in intracranial aneurysms — Comparative velocity investigations with high resolution lattice Boltzmann simulations, normal resolution ANSYS simulations, and MR imaging. *Med. Phys.* **43**, 6186–6198 (2016).

82. Bai-Nan, X., Fu-Yu, W., Lei, L., Xiao-Jun, Z. & Hai-Yue, J. Hemodynamics model of fluid-solid interaction in internal carotid artery aneurysms. *Neurosurg. Rev.* **34**, 39–47 (2011).

83. Voß, S., Beuing, O., Janiga, G. & Berg, P. Multiple aneurysms anatomy challenge 2018 (MATCH)—phase Ib: effect of morphology on hemodynamics. *PLoS One* **14**, e0216813 (2019).

84. Saho, T. & Onishi, H. Quantitative comparison of hemodynamics in simulated and 3D angiography models of cerebral aneurysms by use of computational fluid dynamics. *Radiol. Phys. Technol.* **8**, 258–265 (2015).

85. Maas, S. A., Ellis, B. J., Ateshian, G. A. & Weiss, J. A. FEBio: Finite Elements for Biomechanics. *J. Biomech. Eng.* **134**, (2012).

86. Madhavan, S. & Kemmerling, E. M. C. The effect of inlet and outlet boundary conditions in image-based CFD modeling of aortic flow. *Biomed. Eng. Online* **17**, (2018).

87. Xu, P. *et al.* Assessment of boundary conditions for CFD simulation in human carotid artery. *Biomech. Model. Mechanobiol.* **17**, 1–17 (2018).

88. Canchi, T., Kumar, S. D., Ng, E. Y. K. & Narayanan, S. A Review of Computational Methods to Predict the Risk of Rupture of Abdominal Aortic Aneurysms. *BioMed Research International* **2015**, (2015).

89. Valencia, A. *et al.* Fluid Structural Analysis of Human Cerebral Aneurysm Using Their Own Wall Mechanical Properties. *Comput. Math. Methods Med.* **2013**, 293128 (2013).

90. Cebral, J. R. *et al.* Combining data from multiple sources to study mechanisms of aneurysm disease: Tools and techniques. *Int. j. numer. method. biomed. eng.* **34**, (2018).

91. Mut, F. *et al.* Computational hemodynamics framework for the analysis of cerebral aneurysms. *Int. j. numer. method. biomed. eng.* **27**, 822–839 (2011).

92. Longo, M. *et al.* Role of hemodynamic forces in unruptured intracranial aneurysms: an overview of a complex scenario. *World Neurosurg.* **105**, 632–642 (2017).

93. Meng, H., Tutino, V. M., Xiang, J. & Siddiqui, A. High WSS or Low WSS? Complex interactions of hemodynamics with intracranial aneurysm initiation, growth, and rupture: toward a unifying hypothesis. *Am. J. Neuroradiol.* **35**, 1254–1262 (2014).

94. Munarriz, P. M. *et al.* Basic principles of hemodynamics and cerebral aneurysms. *World Neurosurgery* **88**, 311–319 (2016).

95. Reza, M. M. S. & Arzani, A. A critical comparison of different residence time measures in aneurysms. *J. Biomech.* **88**, 122–129 (2019).

96. Esmaily-Moghadam, M., Hsia, T.-Y. & Marsden, A. L. A non-discrete method for computation of residence time in fluid mechanics simulations. *Phys. fluids* **25**, 110802 (2013).
